# Predatory mite instars (Acari, Mesostigmata) and decomposing tree leaves in mixed and monoculture stands growing on a spoil heap and surrounding forests

**DOI:** 10.1007/s10493-021-00646-y

**Published:** 2021-07-26

**Authors:** Cezary K. Urbanowski, Paweł Horodecki, Jacek Kamczyc, Maciej Skorupski, Andrzej M. Jagodziński

**Affiliations:** 1 Department of Game Management and Forest Protection, Poznań University of Life Sciences, Wojska Polskiego 71C, 60-625 Poznań, Poland; 2 Institute of Dendrology, Polish Academy of Sciences, Parkowa 5, 62-035 Kórnik, Poland

**Keywords:** Post-mining site, Afforestation, Soil reclamation, Organic matter, Developmental stages, Mite assemblages

## Abstract

In the past, ecological research mainly omitted the sexual and developmental variability of mite communities, and therefore could not fully reflect the actual state and function of mite communities in the ecosystems studied. The aim here was to analyze how habitat conditions (mixed vs. monoculture stands) and single-species litter of 14 tree species (in mixed stands) affect the sex and developmental stages of Mesostigmata mites living on the decomposing litter. The research was conducted in 2011–2016, at the Bełchatów Lignite Mine external spoil heap (Central Poland) in mixed stands growing on the spoil heap, as well as in pine and birch monoculture stands growing on the spoil heap and an adjacent forest area. We found significant influences of habitat on females, males and juveniles. Additionally, we found that soil mean temperature had a significant effect on males and juveniles, but not on females. Moreover, despite the insignificant influence of litter species on mite communities, we found that percentage litter mass loss significantly affected female and juvenile mites. Taking into account habitat type, the percentage litter mass loss significantly affected female and male mites, but not juveniles. The mite abundance calculated per dry litter mass usually gradually increased during decomposition. Interestingly, the highest mean female, male and juvenile abundances were recorded in birch stands growing on the adjacent forest area; however, juvenile mites were also very numerous in mixed stands on spoil heap. Therefore, our results confirm that mixed stands on post-mining areas are a potentially better habitat for development of mesostigmatid communities compared to monocultures, among others by relatively higher humidity and lower temperatures.

## Introduction

The decomposition process, the physicochemical breakdown of organic plant matter, is one of the most important processes occurring in nature (Field et al. 1998; Schulze 2000; Graca et al. 2005; Berg and McClaugherty 2014; Eisenhauer et al. 2017; Fujii and Takeda 2017). Decomposition is regulated by many natural factors, including habitat conditions (Horodecki and Jagodziński 2019) and soil organisms (Urbanowski et al. 2018, 2021; Horodecki et al. 2019), and it still requires more scientific attention on habitats transformed by anthropogenic activity (Berg and McClaugherty 2014; Eisenhauer et al. 2017; Horodecki et al. 2019).

Soil fauna can be directly related to the decomposition of litter organic matter (Fujii and Takeda 2017); however, soil predators that feed on soil organisms can be indirectly involved in the decomposition of litter (Vreeken-Buijs and Brussaard 1996; Lawrence and Wise 2000, 2004; Urbanowski et al. 2018, 2021). One of the most numerous microarthropod groups living in the topsoil is mesostigmatid mites (Koehler 1997, 1999). The majority of mesostigmatid mites are predators that mainly feed on many groups of soil animals (Koehler 1999; Wissuwa et al. 2012; Skorupski et al. 2013). Their community structure, due to high abundance, high susceptibility to environmental factors and occupation of higher trophic levels, makes them valuable bioindicators (Madej 2004; Beaulieu and Weeks 2007; Skorupski et al. 2013).

Previous studies focused on species richness and community structure; however, this topic omitted the changes of population structure (sex ratio and instar proportion), and therefore could not fully reflect the actual population state as well as its function in the ecosystems studied. Changes of population structure may strongly affect the ecosystem functions performed by species forming a community and significantly change the processes taking place in a given ecosystem (Rudolf and Rasmussen 2013). These changes are also noticeable among communities of edaphic fauna, including soil mites. Properly preserved structure of soil mite communities, especially predators, is important for ecosystem balance, as it determines the continuity of these communities over time (Walter and Proctor 2013; FAO 2020). The mite assemblages are influenced by many external factors, not only natural, but also anthropogenic (Madej 2002; Skubała 2002b; Rudolf and Rasmussen 2013). Soil mite communities are also affected by external interactions with other species and soil organisms (Walter and Proctor 2013; Frouz 2018). Such relationships are willingly studied and relatively well-known (Mwabvu 2014; Mueller et al. 2016; Brückner et al. 2017; Seniczak et al. 2018; Frouz 2018), while less attention has been given to intra-population relationships and community structure (Rudolf and Rasmussen 2013), including developmental stages of mites (Seniczak et al. 2014, 2018; Salmane and Spuņģis 2015; Urbanowski et al. 2018, 2021). A better understanding of the sex and developmental structure of soil mite communities could contribute to a better understanding of soil biodiversity and ecological functions of soil mites. This topic seems even more important in the context of soil revitalization on areas transformed by human activity.

The developmental instars of mesostigmatid mites include adult (males, females) and three juvenile instars (deutonymphs, protonymphs and larvae) (Giljarov and Bregetova 1977; Gwiazdowicz 2007). The life strategy of gamasid mites depends on their developmental stage due to differences in the optimal living conditions and adaptability to the environment (Huhta and Hänninen 2001). Sclerotization and body structure affect water loss from the mite body, as well as their susceptibility to insolation and fluctuations of environmental factors (Madej 2002; Skubała 2002b). Thus, more sclerotized and larger adult developmental stages should be more resistant to water loss, which makes them less susceptible to changes of climatic temperature and humidity compared to juvenile mites. Therefore, we may expect differences in proportional abundance of adults and juvenile instars in various habitats, including decomposed litter.

Some studies focused also on sex ratio and life strategy of Mesostigmata, including parthenogenesis (Norton et al. 1993; Błoszyk et al. 2004). Mites, including Mesostigmata, are highly diverse in terms of the reproduction types (Bogdanowicz et al. 2008). For example, Błoszyk et al. (2004) stated that many mesostigmatid mite taxa are parthenogenetic species. Moreover, they revealed that mesostigmatid mite taxa which reproduced without males were associated with forest litter and soil, whereas bisexual species with relatively unstable microenvironments. Also, Norton et al. (1993) provides a great outline of different reproduction types for soil Mesostigmata, which affect mesostigmatid sex ratio.

Recognition of the details of mite development should be used not only to understand the biology and ecology of economically important mesostigmatid species (Azevedo et al. 2018; Roth et al. 2020; Oliveira et al. 2020), but also to create a better bioindication tool based on ecology and biology of individual mesostigmatid species (Urbanowski et al. 2018, 2021). Therefore, the aim of our research was to analyze how different habitat conditions (habitat type experiment; mixed stands vs. monoculture stands) and single litter types (single litter species; litter type experiment conducted in mixed stands) affects the developmental stages (females, males and juvenile instars) of Mesostigmata mite communities living in the decomposing litter in monoculture stands of silver birch (SHbirch) and Scots pine (SHpine), and mixed stands (SHmixed) on an external post-mining spoil heap, as well as in adjacent monoculture stands of silver birch (FHbirch) and Scots pine (FHpine) on forest land.

The availability of micro- and macro-elements contained in the soil is often very limited for soil organisms (Chaturvedi and Singh 2017; Sako et al. 2018). The microhabitat conditions of the topsoil significantly shape the soil fauna communities through the availability of nutrients (Frouz 2018; Urbanowski et al. 2021). In addition, many studies show that a higher diversity of the stand species composition (mixed stands) increases resistance to extreme climatic factors (Neuner et al. 2015; Vitali et al. 2018; Vacek et al. 2021); moreover, it also has a positive effect on soil fauna when compared with single-species stands (Cavard et al. 2011; Korboulewsky et al. 2016; Urbanowski et al. 2021). Therefore, we hypothesized that (1) habitat conditions (mixed stands vs. monoculture stands; spoil heap vs. forest land) would significantly affect the abundance of each gamasid mite developmental stage. We subsequently hypothesized that abundance of females, males and juvenile instars would be (1A) lower in monoculture stands compared to mixed stands, and (1B) lower on the spoil heap compared to adjacent forest area.

As many studies revealed, mesostigmatid mites feed primarily on herbivorous, fungivorous and saprophagous organisms (Koehler 1999; Wissuwa et al. 2012; Skorupski et al. 2013), which may be directly dependent on fallen litter features (Fujii and Takeda 2017; Kamczyc et al. 2019; Urbanowski et al. 2021). Moreover, microhabitat properties (Frouz 2018), including leaf structure (Schmidt 2014), as well as soil-forming processes, including forest litter decomposition (Osler et al. 2004; Urbanowski et al. 2018, 2021), may significantly shape soil fauna communities, including mesostigmatid mites. Therefore, we hypothesized that (2) the litter species would significantly affect the abundance of each mesostigmatid developmental stage. Finally, we hypothesized that (3) as litter decomposition progressed with collection date, the abundance of females, males and juveniles would differ significantly among collection dates.

## Material and methods

### Site description and experimental design

The research was established in 2011–2016 at the external spoil heap (Bełchatów Lignite Mine, Mount Kamieńsk, Central Poland; 51°12′54″N, 19°26′04″E). The spoil heap was formed to a relative height of approx. 190 m above the level of the surrounding forest land in the years 1977–1993 (Pietrzykowski 2010; Rawlik et al. 2018a). The total area of the spoil heap is 1471 ha and today it is the largest facility of its type in Central and Eastern Europe (Jagodziński et al. 2018). The most common soils on the spoil heap toplayer are Quaternary loamy, gravelly sands with sporadic occurrences of loams and clays. Additionally, the soil contains tertiary sands with high acidity (pH < 4.5) with loam and clay inclusions (Pietrzykowski and Daniels 2014). The soil substrate is very poor in organic matter, additionally there is a toxic tertiary substrate on the top in some places (Jagodziński et al. 2018).

Weather conditions were described on the basis of the nearest meteorological station (51°43′19″N, 19°23′53″E, 179 m a.s.l.). The average precipitation and temperature in the study area were 665 mm and 8.65 °C, respectively (data during 1995–2015), whereas the growing season lasts from 210 to 220 days per calendar year (Jagodziński et al. 2018). The undergrowth vegetation on the spoil heap plots was composed mainly of *Calamagrostis epigejos* (L.) Roth, *Festuca pratensis* Huds. and *Festuca rubra* L. (Rawlik et al. 2018a, b), whereas the undergrowth on research plots located on the adjacent forest area was composed of *Carex ovalis* Good., *Vaccinium myrtillus* L. and *Moehringia trinervia* (L.) Clairv. (Urbanowski et al. 2018).

The decomposition experiment began in 2011 and lasted 5 years (2011–2016). All the litter that was inside the litterbags was collected ex-situ during autumn of the same year, but still on the spoil heap area. The litter used was oven-dried and weighed and then placed in litterbags of fiberglass fabric (1 mm mesh size, 15 × 15 cm) on the 11 research plots—three plots in mixed stands on the spoil heap, two plots in monoculture Scots pine stands and two in silver birch stands on the spoil heap, and two plots in monoculture Scots pine stands and two in silver birch stands on the forest area. All samples were randomly laid out in December 2011 and collected after 91, 182, 275, 366, 548, 730, 913, 1100 days in monoculture stands, as well as after 95, 196, 285, 385, 564, 753, 936, 1117, 1298 and 1754 days in mixed stands. Single-species stands growing on the spoil heap and forest areas were covered by *Pinus sylvestris* L. and *Betula pendula* Roth, wherea all mixed stands were covered mostly by *B. pendula*, *Larix decidua* Mill., *Pinus nigra* Arn., *P. sylvestris* and *Robinia pseudoacacia* L. (for more basic plot characteristics see Urbanowski et al. 2018, 2021). In mixed stands litterbags contained single-species litter of *Acer pseudoplatanus* L., *A. glutinosa*, *B. pendula*, *Fagus sylvatica* L., *Fraxinus excelsior* L., *P. sylvestris*, *Populus nigra* L. ‘Italica’, *Populus tremula* L., *Populus* × *canadensis* Moench, *Prunus serotina* Ehrh., *Quercus robur* L., *Quercus rubra* L., *R. pseudoacacia* and *Ulmus laevis* Pall., whereas in monoculture stands: *Alnus glutinosa* (L.) Gaertn., *B. pendula*, *P. sylvestris* and *Q. robur*, and only these four litter species were analized in the mixed stands in the habitat type experiment. In total, 2413 litterbag samples were laid out; however, as a result of random external factors (e.g., litterbag destruction by wildlife), 28 samples were excluded from further analysis. After sampling, litterbags were transported to the Department of Game Management and Forest Protection laboratory (Poznań University of Life Sciences, Poznań, Poland) in order to isolate the soil fauna (see below), then oven-dried at 65 °C to a constant dry mass to estimate the initial dry litter mass and percentage litter mass loss. Additionally, on the research plots in mixed stands, the soil mean temperature was measured using HOBO U23 Pro v.2 data loggers (1-h measurement intervals; located around 3 cm beneath the mineral soil surface).

### Mesostigmata investigation

Soil fauna was extracted from litterbags using Berlese-Tullgren funnels (2 mm mesh size) for a minimum of 6 days (constant temperature in funnels near samples: 30 ± 0.5 °C), until the collected samples were dry. The changing moisture and temperature gradient in the funnels forced soil fauna to migrate directly down to the containers with 75% ethyl alcohol. After the extraction process, mesostigmatid mites were selected using a stereomicroscope, then mounted on slides in 85% lactic acid for a minimum of 3 days to ensure maximum transparency of selected specimens. Afterwards, mites were identified to the genus and/or species level, including developmental stages, using acarological keys (i.e., Micherdziński 1969; Karg 1971, 1993; Giljarov and Bregetova 1977; Gwiazdowicz 2007).

### Statistical analysis

To keep comparability, all integer values were calculated to mean values followed by the standard deviation (SD). We calculated the mean number of mite specimens per plot, litter type and collection date to reduce the number of replications per variant. Mean mite abundance was calculated per 100 g of dry litter mass. To compare the mutual relationships of mite developmental stages (females per male; juveniles per female) between habitat and litter types we used the *HSD.test* function from the *agricolae* package (de Mendiburu 2013). The mutual relationships were calculated based on total abundance. Furthermore, we analyzed ‘collection date’ and ‘plot selection’ as random effects. Models (backward model selection) were developed using the *lme4* package (Bates et al. 2015). We applied predictors with low variance inflation factors (VIF < 3). Moreover, as was supported by overdispersion which can be detected by dividing the residual deviance by the degrees of freedom, we applied negative binomial distributions for each developmental stage and used generalized linear mixed models (GLMM). Moreover, we also included a quadratic term for soil mean temperature in the litter type experiment to allow for a nonlinear relationship in models of mean abundance of males and juveniles, as supported by the improvement of the model AICc scores (males, AICc = -1.04; juveniles, AICc = − 1.84). The statistical significance of variables used in models (habitat type, litter type, collection date, percentage litter mass loss and soil mean temperature) was calculated using z-values, implemented in the *lmerTest* package (Kuznetsova et al. 2017). Additionally, Tukey post-hoc tests were done to determine the differences between the litter and habitat types studied. Statistical analyses were done using R software (https://www.Rproject.org/).

## Results

In total (habitat and litter type experiments), we recorded 25,763 mites indicated as 81 taxa (77 species and four genera; Appendix 1). Moreover, generalized linear mixed models (assuming negative binomial distributions) explaining mean female, male and juvenile abundances per dry litter mass within experiments indicated no statistical influence of collection date on any mite developmental stage (Table 1).

**Table 1:** Generalized linear mixed models assuming negative binomial distribution explaining mean female, male and juvenile abundances per 100 g dry litter mass within experiments

Response	Term	Estimate	SE	z	Pr( >|z|)
HTexp					
Females	(Intercept)	4.4931	0.6859	6.5510	< 0.0001
	FHpine	− 1.3923	0.3790	− 3.6740	< 0.0001
	SHmixed	− 0.8118	0.5369	− 1.5120	0.13
	SHbirch	− 1.8930	0.3878	− 4.8810	< 0.0001
	SHpine	− 3.1642	0.3919	− 8.0740	< 0.0001
	plml	0.0269	0.0096	2.8020	0.0051
	Random effect—collection date	Variance	0.8931	SD	0.9451
	Random effect—plot	Variance	< 0.0001	SD	< 0.0001
Males	(Intercept)	3.4497	0.5933	5.8140	< 0.0001
	FHpine	− 1.4147	0.4260	− 3.3210	< 0.0001
	SHmixed	− 1.8366	0.4794	− 3.8310	< 0.0001
	SHbirch	− 2.3448	0.4620	− 5.0760	< 0.0001
	SHpine	− 2.8060	0.4472	− 6.2750	< 0.0001
	plml	0.0176	0.0084	2.0890	0.037
	Random effect—collection date	Variance	0.4501	SD	0.6709
	Random effect—plot	Variance	< 0.0001	SD	< 0.0001
Juveniles	(Intercept)	4.4240	0.7844	5.6400	< 0.0001
	FHpine	− 1.1846	0.4978	− 2.3800	0.017
	SHmixed	− 0.7614	0.6397	− 1.1900	0.23
	SHbirch	− 3.9030	0.5201	− 7.5040	< 0.0001
	SHpine	− 4.0396	0.5212	− 7.7510	< 0.0001
	plml	0.0207	0.0109	1.9030	0.057
	Random effect—collection date	Variance	1.0271	SD	1.0135
	Random effect—plot	Variance	0.0618	SD	0.2486
Ltexp					
Females	(Intercept)	3.8179	0.4965	7.6900	< 0.0001
	Aln_glu	− 0.3666	0.2484	− 1.4760	0.14
	Bet_pen	− 0.1332	0.2501	− 0.5330	0.59
	Fag_syl	− 0.3480	0.2777	− 1.2530	0.21
	Fra_exc	− 0.1093	0.2488	− 0.4390	0.66
	Pin_syl	− 0.2590	0.2613	− 0.9910	0.32
	Pop_can	− 0.0426	0.2504	− 0.1700	0.86
	Pop_nig	− 0.4801	0.2582	− 1.8590	0.063
	Pop_tre	− 0.0233	0.2477	− 0.0940	0.93
	Pru_ser	− 0.3116	0.2565	− 1.2150	0.22
	Que_rob	− 0.5071	0.2862	− 1.7720	0.076
	Que_rub	− 0.1852	0.2583	− 0.7170	0.47
	Rob_pse	− 0.2245	0.2628	− 0.8540	0.39
	Ulm_lae	0.3933	0.2529	1.5550	0.12
	smt	0.0651	0.0364	1.7880	0.074
	plml	0.0174	0.0043	4.0080	< 0.0001
	Random effect—collection date	Variance	0.2393	SD	0.4891
	Random effect—plot	Variance	0.0893	SD	0.2989
Males	(Intercept)	1.2093	0.5662	2.1360	0.033
	Aln_glu	− 0.6540	0.5078	− 1.2880	0.20
	Bet_pen	− 0.3192	0.4995	− 0.6390	0.52
	Fag_syl	− 0.2400	0.5221	− 0.4600	0.65
	Fra_exc	− 0.2935	0.5101	− 0.5750	0.57
	Pin_syl	− 0.7689	0.5064	− 1.5180	0.13
	Pop_can	− 0.2710	0.5101	− 0.5310	0.60
	Pop_nig	− 0.9187	0.5077	− 1.8100	0.070
	Pop_tre	0.1968	0.5149	0.3820	0.70
	Pru_ser	− 0.0965	0.5250	− 0.1840	0.85
	Que_rob	− 0.8703	0.5253	− 1.6570	0.098
	Que_rub	− 0.7701	0.5111	− 1.5070	0.13
	Rob_pse	− 0.2462	0.5094	− 0.4830	0.63
	Ulm_lae	− 0.2629	0.5157	− 0.5100	0.61
	smt	0.3264	0.1301	2.5100	0.012
	smt^2	− 0.0148	0.0073	− 2.0290	0.042
	plml	0.0097	0.0050	1.9440	0.052
	Random effect—collection date	Variance	0.0509	SD	0.2257
	Random effect—plot	Variance	< 0.0001	SD	< 0.0001
Juveniles	(Intercept)	1.2617	0.6314	1.9980	0.046
	Aln_glu	− 0.3039	0.3255	− 0.9340	0.35
	Bet_pen	− 0.6707	0.3246	− 2.0660	0.039
	Fag_syl	− 0.6718	0.3576	− 1.8790	0.060
	Fra_exc	− 0.4426	0.3240	− 1.3660	0.17
	Pin_syl	− 0.3378	0.3356	− 1.0060	0.31
	Pop_can	− 0.1222	0.3255	− 0.3750	0.71
	Pop_nig	− 0.3990	0.3388	− 1.1780	0.24
	Pop_tre	0.1984	0.3269	0.6070	0.54
	Pru_ser	− 0.0966	0.3326	− 0.2910	0.77
	Que_rob	− 0.6927	0.3651	− 1.8970	0.058
	Que_rub	− 0.4820	0.3314	− 1.4550	0.15
	Rob_pse	− 0.5932	0.3368	− 1.7610	0.078
	Ulm_lae	− 0.0045	0.3243	− 0.0140	0.99
	smt	0.5124	0.1336	3.8370	< 0.0001
	smt^2	− 0.0183	0.0076	− 2.4120	0.016
	plml	0.0197	0.0049	3.9870	< 0.0001
	Random effect—collection date	Variance	0.1181	SD	0.3436
	Random effect—plot	Variance	0.4708	SD	0.6861

*HTexp* habitat type experiment, *LTexp* litter type experiment, *plml* percentage litter mass loss, *smt* soil mean temperature

### Mite instars among habitat types

In the habitat type experiment we found 12,145 specimens (6395 females, 855 males and 4895 juveniles). For each developmental stage we found that habitat type significantly affected mite assemblages. Moreover, taking into account habitat type, the percentage litter mass loss also significantly affected mite assemblages, except for juvenile stages (Table 1).

The highest mean female abundance was found in FHbirch (550.09 ± 444.14 specimens), whereas the lowest was found in SHpine (24.10 ± 25.55). Mean female abundance differed significantly between those habitats (Tukey test: P < 0.0001). Interestingly, the mean female abundance recorded in SHmixed (217.66 ± 237.38 specimens) differed significantly only from SHpine (Tables 2 and 3). The mean female abundance was highest at 1100 days after the start of the experiment in FHbirch (1204.44 ± 416.25 specimens), on day 730 in SHbirch (317.28 ± 309.24), and on day 1754 in SHmixed (740.41 ± 471.20). The highest mean female abundances in monoculture pine stands were recorded on day 275 (FHpine, 446.40 ± 16.52; SHpine, 70.30 ± 10.84) (Appendix 2).

**Table 2:** Tukey post-hoc tests conducted for habitat type in models assuming negative binomial distribution for mean female, male and juvenile abundances calculated per 100 g dry litter mass

Response	Term	Estimate	SE	Df	z ratio	p
Females	FHbirch – FHpine	1.392	0.379	Inf	3.674	0.0024
	FHbirch – SHbirch	1.893	0.388	Inf	4.881	< 0.0001
	FHbirch – SHpine	3.164	0.392	Inf	8.074	< 0.0001
	FHpine – SHpine	1.772	0.351	Inf	5.046	< 0.0001
	SHmixed – SHpine	2.352	0.552	Inf	4.260	0.0002
	SHbirch – SHpine	1.271	0.339	Inf	3.745	0.0018
Males	FHbirch – FHpine	1.415	0.426	Inf	3.321	0.0090
	FHbirch – SHmixed	1.837	0.479	Inf	3.831	0.0013
	FHbirch – SHbirch	2.345	0.462	Inf	5.076	< 0.0001
	FHbirch – SHpine	2.806	0.447	Inf	6.275	< 0.0001
	FHpine – SHpine	1.391	0.411	Inf	3.388	0.0070
Juveniles	FHbirch – SHbirch	3.903	0.520	Inf	7.504	< 0.0001
	FHbirch – SHpine	4.04	0.521	Inf	7.751	< 0.0001
	FHpine – SHbirch	2.718	0.494	Inf	5.500	< 0.0001
	FHpine – SHpine	2.855	0.487	Inf	5.860	< 0.0001
	SHmixed – SHbirch	3.142	0.660	Inf	4.762	< 0.0001
	SHmixed – SHpine	3.278	0.662	Inf	4.952	< 0.0001

*FHbirch* monoculture stands of silver birch growing on forest habitat, *FHpine* monoculture stands of Scots pine growing on forest habitat, *SHmixed* mixed stands growing on spoil heap, *SHbirch* monoculture stands of silver birch growing on spoil heap, *SHpine* monoculture stands of Scots pine growing on spoil heap, *Inf* infinity

**Table 3:** Mean (± SD) female, male and juvenile abundances per 100 g dry litter mass recorded within habitat types

Habitat type	Females	Males	Juveniles
FHbirch	550.09 ± 444.14	90.44 ± 86.89	303.02 ± 211.02
FHpine	136.10 ± 147.83	23.16 ± 27.44	98.16 ± 91.42
SHmixed	217.66 ± 237.38	16.19 ± 15.31	212.83 ± 269.76
SHbirch	119.59 ± 155.74	10.5 ± 18.74	8.22 ± 14.82
SHpine	24.10 ± 25.55	6.47 ± 7.75	8.13 ± 14.48

*FHbirch* monoculture stands of silver birch growing on forest habitat, *FHpine* monoculture stands of Scots pine growing on forest habitat, *SHmixed* mixed stands growing on spoil heap, *SHbirch* monoculture stands of silver birch growing on spoil heap, *SHpine* monoculture stands of Scots pine growing on spoil heap

The mean male abundances were highest in FHbirch (90.44 ± 86.89 specimens), and lowest in SHpine (6.47 ± 7.75). Mean male abundance recorded in FHbirch differed significantly from each habitat type examined in terms of mean male abundance (Tables 2 and 3). The mean male abundances were highest 730 days after the experiment started on the forest area (FHbirch, 227.06 ± 123.42; FHpine, 58.70 ± 51.51). On the spoil heap area the highest values of mean male abundance were recorded after 275 days (SHbirch, 53.20 ± 27.97; SHpine, 21.88 ± 1.19). In SHmixed the highest values of mean male abundance were obtained after 1754 days (44.70 ± 30.13) at the end of experiment (Appendix 2).

The mean juvenile abundance was highest in FHbirch (303.02 ± 211.02 specimens). However, we also recorded high mean abundance of juvenile mites in SHmixed (212.83 ± 269.76), when compared to the lowest values recorded in SHbirch (8.22 ± 14.82) and SHpine (8.13 ± 14.48). Mean juvenile abundances recorded in FHpine (98.16 ± 91.42), as well as FHbirch and SHmixed, differed significantly from monoculture stands growing on the spoil heap (P < 0.0001) (Tables 2 and 3). The mean juvenile abundance was highest on day 1100 in FHbirch (721.93 ± 94.03). On the same day we recorded the highest value of mean juvenile abundance in FHpine (213.10 ± 25.72). On the spoil heap area, the juvenile mites reached the highest mean abundances after 275 days in SHbirch (43.55 ± 16.81) and SHpine (40.02 ± 15.25), as well as after 936 days in SHmixed (574.36 ± 422.52) (Appendix 2).

The analysis of mutual instar relationships showed that the mean number of juvenile instars per female was the lowest in SHbirch (0.09 ± 0.14). Furthermore, the mean number of females per male was the lowest in SHpine (2.46 ± 2.36); however, the mean number of females per male was similar on each habitat type studied (Tukey test: P > 0.05). Moreover, the mean number of juvenile instars per female differed significantly between habitat types examined (Table 4).

**Table 4:** Mutual relationships of mite developmental stages recorded within habitat types (data presented as mean values ± SD)

Habitat type	Females per male	Females/male [%]	Juveniles per female	Juveniles/female [%]
FHbirch	13.18 ± 12.54a	81.37 ± 23.77ab	0.74 ± 0.44abc	39.61 ± 12.49a
FHpine	9.31 ± 16.53a	69.53 ± 36.06ab	0.93 ± 1.08ab	40.22 ± 31.89a
SHmixed	12.39 ± 8.23a	92.19 ± 4.94a	0.94 ± 0.89a	39.42 ± 22.30a
SHbirch	11.27 ± 17.32a	58.74 ± 43.17b	0.09 ± 0.14c	13.35 ± 25.09b
SHpine	2.46 ± 2.36a	61.25 ± 37.55b	0.30 ± 0.35bc	18.51 ± 18.89ab

*FHbirch* monoculture stands of silver birch growing on forest habitat, *FHpine* monoculture stands of Scots pine growing on forest habitat, *SHmixed* mixed stands growing on spoil heap, *SHbirch* monoculture stands of silver birch growing on spoil heap, *SHpine* monoculture stands of Scots pine growing on spoil heap

Means within a column followed by different letters are significantly different (Tukey post hoc test: P < 0.05)

The analysis of mite developmental stages on the species-level for the most dominant mite species revealed that juveniles of *Trachytes aegrota* recorded in SHmixed constituted the majority of individuals of this mite species (55.1% of total abundance). Also abundant were females of this mite species in the same habitat type (26.7% of total abundance). Interestingly, females and juveniles of *Veigaia nemorensis* and *Zercon peltatus* were the most abundant in forest areas. Equally numerous occurrences of females and juveniles of *V. nemorensis* were also recorded in SHmixed (females, 24.0% of total abundance; juveniles, 11.9%) (Table 5).

**Table 5:** Mite developmental stages share [%] on the species-level for the most dominant mite species (see Appendix 1) recorded within habitat types (data based on total abundance of a given mite species)

Habitat type	Mite species		
	*Trachytes aegrota*	*Zercon peltatus*	*Veigaia nemorensis*
FHbirch			
Females	8.96	22.30	31.03
Males	0	14.07	0
Juveniles	8.27	9.60	24.78
FHpine			
Females	0.38	29.40	3.59
Males	0	2.28	0
Juveniles	0.46	21.56	4.60
SHmixed			
Females	26.74	0.04	23.99
Males	0	0	0
Juveniles	55.09	0.00	11.93
SHbirch			
Females	0	0.18	0
Males	0	0	0
Juveniles	0	0.13	0
SHpine			
Females	0	0.22	0
Males	0	0.04	0
Juveniles	0.10	0.18	0.07

*FHbirch* monoculture stands of silver birch growing on forest habitat, *FHpine* monoculture stands of Scots pine growing on forest habitat, *SHmixed* mixed stands growing on spoil heap, *SHbirch* monoculture stands of silver birch growing on spoil heap, *SHpine* monoculture stands of Scots pine growing on spoil heap

### Mite instars among litter types

In the litter type experiment we found 19,296 specimens (9198 females, 832 males, 9266 juveniles). Interestingly, the generalized linear mixed model that explained mean juvenile abundance per dry litter mass indicated a significant influence of litter type. Furthermore, percentage litter mass loss significantly affected the mean abundances of female and juvenile developmental stages. Also, the soil mean temperature had a significant effect on mean male and juvenile abundances, but not on females (Table 1). Tukey tests conducted for the litter type experiment in models (assuming negative binomial distributions) for mean female, male and juvenile abundances calculated per 100 g dry litter mass indicated that there were no significant differences in each developmental stage.

The highest mean female abundance was found in *U. laevis* litter (522.75 ± 859.15 specimens), whereas the lowest was in *Q. robur* (117.93 ± 132.64) (Table 6). The highest mean female abundances for each litter type were found after 1754 days (10 litter types) and 1298 days (three litter types) since the experiment started. Only in *P. serotina* litter (722.34 ± 1190.74) was the mean female abundance highest after 1117 days. Interestingly, there were no female mites in *P. serotina* after 936 days. On the same day the lowest mean female abundance for *P.* × *canadensis* litter (14.11 ± 6.31) was recorded; however, the remainder of litter types had the lowest mean female abundances at the first collection date, 95 days since the experiment started (Appendix 3).

**Table 6:** Mean (± SD) female, male and juvenile abundances per 100 g dry litter mass recorded within 14 litter types

Litter type	Females	Males	Juveniles
Ace_pse	305.99 ± 250.89	30.68 ± 52.68	354.87 ± 472.48
Aln_glu	255.32 ± 331.24	19.35 ± 44.27	314.17 ± 533.62
Bet_pen	277.72 ± 536.41	20.15 ± 24.46	181.89 ± 296.26
Fag_syl	120.15 ± 93.52	16.61 ± 21.31	98.61 ± 106.43
Fra_exc	300.81 ± 222.18	20.48 ± 31.70	300.47 ± 459.92
Pin_syl	165.95 ± 115.50	12.53 ± 15.84	179.71 ± 192.17
Pop_can	344.83 ± 736.88	18.86 ± 51.40	238.55 ± 451.66
Pop_nig	154.87 ± 144.44	10.54 ± 12.40	144.73 ± 155.81
Pop_tre	319.74 ± 432.16	53.66 ± 208.96	512.00 ± 1083.18
Pru_ser	271.79 ± 427.07	31.87 ± 93.60	499.81 ± 1260.48
Que_rob	117.93 ± 132.64	9.99 ± 15.40	118.05 ± 163.26
Que_rub	192.36 ± 168.18	11.89 ± 13.23	153.54 ± 162.67
Rob_pse	160.58 ± 122.64	19.58 ± 20.27	124.21 ± 126.55
Ulm_lae	522.75 ± 859.15	20.34 ± 35.33	330.68 ± 399.59

Litter types are abbreviated to the first three letters of genera name and first three letters of the species name

The highest mean male abundance was found in *P. tremula* litter (53.66 ± 208.96 specimens), whereas the lowest was in *Q. robur* (9.99 ± 15.40) (Table 6). The highest mean male abundances for each litter type were found at 1754 (five litter types), 1298 (three litter types) and 1117 days (four litter types) since the experiment started. In *F. sylvatica* litter, the highest mean male abundance (38.97 ± 22.28) was recorded after 285 days, whereas for *U. laevis* (78.05 ± 85.95) it was recorded after 936 days. The mean male abundance was highest at day 1754 in *P. tremula* litter (383.14 ± 663.62), whereas there were no male mites at the first collection date (day 95) in *P. nigra*, *P. serotina* and *Q. rubra* litter. Likewise, there were no male mites in *A. pseudoplatanus* (day 1754), *A. glutinosa* (936, 1117 and 1298), *F. excelsior* (1117, 1754), *P.* × *canadensis* (936, 1298), *P. serotina* (936, 1298), *Q. rubra* (753), and *U. laevis* litter (1298) (Appendix 3).

The highest mean juvenile abundance was found in *P. tremula* litter (512.00 ± 1083.18 specimens), whereas the lowest was in *F. sylvatica* (98.61 ± 106.43). Interestingly, in *P. serotina* litter we also found very high mean juvenile abundance (499.81 ± 1260.48), when compared to other litter types examined (Table 6). The highest mean values of juvenile abundance for each litter type were found on days 936 (five litter types), 1117 (three litter types), 564 (two litter types) and 196 (two litter types) since the experiment started. Moreover, in *Q. rubra* (305.40 ± 129.60) and *U. laevis* litter (761.91 ± 681.43) we recorded the highest mean juvenile abundances at 1298 and 1754 days, respectively. The mean juvenile abundance was highest on day 1117 in *P. serotina* litter (2172.53 ± 3762.94). All the litter types examined had the lowest mean juvenile abundances on the first collection date (day 95); however, the lowest were recorded on day 95 in *B. pendula* (1.21 ± 2.09) and *Q. robur* litter (1.59 ± 2.76) (Appendix 3).

The analysis of mutual instar relationships showed that the mean number of females per male was highest in *P. sylvestris* litter (9.08 ± 10.53 specimens). The lowest value was recorded in *P. serotina* (3.32 ± 5.77). However, an equally high value of the mean number of females per male was recorded in *R. pseudoacacia* litter (9.08 ± 9.18). Moreover, we found that the highest number of juveniles per female was in *A. glutinosa* litter (1.66 ± 3.87), whereas the lowest was in *B. pendula* (0.71 ± 0.83). Tukey tests conducted for mutual instar relationships, indicated no significant differences among litter types. The percentage of females to males for each litter type was > 75 (78.4 ± 36.5–92.7 ± 7.9%), whereas the percentage of juveniles to females remained stable (34.7 ± 25.9–45.6 ± 28.9%) (Table 7).

**Table 7:** Mutual relationships of mite developmental stages recorded within litter types (data presented as mean values ± SD)

Litter type	Females per Male	Females/Male [%]	Juveniles per Female	Juveniles/Female [%]
Ace_pse	8.60 ± 14.22	86.32 ± 23.30	1.26 ± 1.50	41.00 ± 27.20
Aln_glu	7.62 ± 11.44	85.67 ± 29.77	1.66 ± 3.87(a)	39.17 ± 31.52
Bet_pen	8.13 ± 9.73	87.28 ± 17.98	0.71 ± 0.83	34.71 ± 25.90
Fag_syl	7.19 ± 10.95	86.05 ± 19.64	0.88 ± 0.77	38.13 ± 23.18
Fra_exc	4.56 ± 5.58	85.07 ± 25.73	0.96 ± 1.27	35.00 ± 26.21
Pin_syl	9.08 ± 10.53	92.67 ± 7.93	0.91 ± 0.86	38.06 ± 23.26
Pop_can	7.62 ± 10.54	89.94 ± 19.72	1.24 ± 2.70	36.98 ± 24.50
Pop_nig	6.92 ± 8.07	92.27 ± 8.44	1.52 ± 2.67	39.61 ± 27.98
Pop_tre	7.57 ± 11.90	87.70 ± 21.56	1.02 ± 1.24	40.04 ± 27.75
Pru_ser	3.32 ± 5.77	78.44 ± 36.50	0.86 ± 1.03	36.57 ± 28.88
Que_rob	5.25 ± 7.87	82.27 ± 29.40	1.14 ± 1.57	42.90 ± 31.04
Que_rub	6.86 ± 8.22	91.53 ± 12.20	0.84 ± 1.00	34.76 ± 23.86
Rob_pse	9.08 ± 9.18	84.03 ± 19.86	1.00 ± 1.30	36.44 ± 24.02
Ulm_lae	7.93 ± 7.78	83.84 ± 29.18	0.92 ± 1.10	45.55 ± 28.95

Litter types are abbreviated to the first three letters of genera name and first three letters of the species name

Means within a column were not significantly different (Tukey post hoc test: P > 0.05)

## Discussion

### Successive changes of gamasid instars and habitat influence on mite communities

Various methods have been used in decomposition studies, but the use of litterbags has recently become one of the most common methods (Kampichler and Bruckner 2009; Urbanowski et al. 2018, 2021; Horodecki et al. 2019; Kamczyc et al. 2019). The litterbag method is a minimally invasive method for soil fauna. Litterbags made of fiberglass fabric, which we used in our research, can also affect edaphone communities. The mesh size of litterbags may determine the conditions inside. Litterbags with smaller meshes could be characterized by increased humidity inside the bag and exclude free access of larger soil fauna to the decomposed organic matter contained in the bag (Kampichler and Bruckner 2009). The fact that we have found mites from the Mesostigmata order, both smaller (< 1 mm body length) and larger (> 1 mm) indicates a correctly adopted litterbag experiment methodology (Kampichler and Bruckner 2009; Urbanowski et al. 2018, 2021; Horodecki et al. 2019).

Many studies have shown that environmental conditions (post-mining vs. forest areas) significantly shape soil fauna communities, including mites (Skubała 2002a; Murvanidze et al. 2013; Urbanowski et al. 2018). Moreover, the positive effect of mixed stands on soil fauna is well known (Irmler 2000; Elmer et al. 2004; Cesarz et al. 2007; Cavard et al. 2011; Korboulewsky et al. 2016; Horodecki et al. 2019; Urbanowski et al. 2021). Compared to monocultures, mixed stands are often characterized by greater biodiversity and better conditions (smaller fluctuations of humidity and temperature fluctuations) for the proper development of soil organisms (Cavard et al. 2011; Korboulewsky et al. 2016; Horodecki et al. 2019; Urbanowski et al. 2021). Interestingly, in our results the highest mean female, male and juvenile abundances were recorded in FHbirch. This confirms the results of Skorupski (2010), who found that silver birch plots had the highest number of species and one of the highest abundances of soil Mesostigmata mites among the 14 tree species of a common-garden experiment; furthermore, the abundances were also higher than on the Scots pine plots. Juvenile mites were also highly numerous in SHmixed.

The reproductive abilities are strongly related to environmental factors (Polis and Strong 1996; Błoszyk et al. 2004). Therefore, in unstable environmental conditions, such as post-mining spoil heaps, species inhabiting this environment may try to maximize reproductive effort by increasing abundance of females, which results in a high number of juvenile stages in communities (Polis and Strong 1996; Napierała and Błoszyk 2013). The high abundance of juveniles in unstable habitat conditions may also result from parthenogenetic reproduction of species (Norton et al. 1993; Błoszyk et al. 2004). On the other hand, Błoszyk et al. (2004) stated that in more stable environments, such as forest litter and soil, parthenogenetic mesostigmatid taxa were more abundant compared to bisexual mite taxa which inhabited more temporary microhabitats. We found that females and juveniles of *T. aegrota* in SHmixed constituted the majority of individuals of this mite species. Błoszyk et al. (2004) stated that this species is a common parthenogenetic mite taxon. Madej and Skubała (1996) and Urbanowski et al. (2021) recorded this species frequently on post-industrial areas. However, some authors stated that this mite species is typical for forest areas (Pastwik et al. 2013; Piasta et al. 2015). Interestingly, we found a high percentage share of females and juveniles of *V. nemorensis*, a typically parthenogenetic mite species (Alberti et al. 2010), in forest areas and in mixed stands growing on the spoil heap. The importance of parthenogenetic mite species in the colonization of habitat-diversified areas is still difficult to estimate (Norton et al. 1993; Madej and Skubała 2002; Błoszyk et al. 2004), even more so in post-industrial areas (Madej and Skubała 2002). Nevertheless, our results show that the spoil heap (especially mixed stands) and forest area were characterized by a high abundance of parthenogenetic mesostigmatid species.

The mean abundance recorded for all instars examined was lowest in the SHpine, which supports our assumptions about the positive effect of mixed stands on development of gamasid mite assemblages in comparison to monoculture stands. The number of juvenile instars in relation to older developmental stages determines the stability of the community structure living in a given area and significantly depends on environmental conditions (Miller and Rudolf 2011; Tuka and Solarz 2017); therefore, that the mean juvenile abundance calculated per female was lowest in the SHbirch and SHpine may indicate that mite assemblages in monoculture stands growing on the spoil heap are less stable compared to monoculture stands growing on forest areas and mixed stands growing on the spoil heap.

Our study indicated the general tendency in colonization of litterbags by mesostigmatid mites. The abundance calculated per 100 g dry litter mass usually gradually increased during decomposition—it had lower values at the beginning and higher values at the last collection dates of the experiment. This tendency was particularly strong for juvenile gamasid mites in which the lowest abundances were always recorded at the first collection date, 95 days since the experiment started (except for FHpine). Moreover, taking into account habitat type, the percentage litter mass loss significantly affected female and male mites, but not juvenile stages. Resources are utilized during ongoing decomposition (Horodecki et al. 2019), therefore, there is more food for soil decomposers, and consequently, also for soil mite predators such as Mesostigmata (Koehler 1999; Beaulieu and Weeks 2007; Wissuwa et al. 2012; Hasegawa et al. 2013). Our results, therefore, may also indicate that the introduced litter has the strongest impacts on soil gamasid mites at the end part of ongoing decomposition.

### Impact of litter type on gamasid instars

It is still difficult to show that a specific mite species is associated with a given tree species, or has greater preference for a given litter or microhabitat (Maraun and Scheu 2000), although Skorupski (2010) showed differences in the species composition of soil Mesostigmata mites in a common-garden experiment of 14 tree species. Moreover, litter and decomposition can significantly influence and shape the mite assemblages (Urbanowski et al. 2018, 2021; Kamczyc et al. 2019), which is consistent with our results. Also, Osler et al. (2004) recorded a significant relationship between decomposition and soil fauna, but only after 20% of litter mass loss. As shown by Frouz (2018), communities can also be shaped by the internal structure of assemblages and other soil fauna communities. Furthermore, the structure of leaves is also important. This was strongly stated by Schmidt (2014), who showed that the structure of leaves can significantly affect mite assemblages. However, we recorded insignificant influences of litter type on adult gamasid developmental stages examined, which may suggest that litter type in litterbags is less important for soil gamasid mites than the surrounding environment.

Many studies indicated the negative impact of *P. serotina* on the environment, including its biodiversity (Robakowski et al. 2018; Dyderski and Jagodziński 2019). In Europe, it is an invasive tree species (Dyderski and Jagodziński 2019), which is characterized by relatively fast litter decomposition (Horodecki et al. 2019). Owing to this, it can change not only litter pH (von Wendorff 1952) but also affect the amount of micro- and macro-elements in the topsoil (Lorenz et al. 2004). Interestingly, we recorded high mean abundance of juvenile mites in *P. serotina* litter, therefore, our results may confirm the positive influence on younger developmental stages, but not on adult mites forming soil gamasid communities.

We found that soil mean temperature had a significant effect on male and juvenile mites, but not on gamasid females. This result is consistent with results of Huhta and Hänninen (2001), who showed that some mite species from the Mesostigmata order were significantly dependent on the prevailing ambient temperature. Also, the results of Thakur et al. (2018), indicated that the mites were dependent on environmental conditions such as temperature or humidity. As juvenile mites are less sclerotized than females (Giljarov and Bregetova 1977; Madej 2002; Skubała 2002b), they may be more susceptible to changes in temperature and humidity of the environment. However, some studies confirmed that food availability is more important for mites than appropriate environmental conditions (Fujii and Takeda 2017). Mite males of some gamasid species appear in low numbers, and their presence is seasonal and sporadic, unlike gamasid females (Norton et al. 1993; Błoszyk et al. 2004; Napierała and Błoszyk 2013). This is related to the reproductive strategy of mesostigmatid mite species, among which there is not only sexual, but also parthenogenetic reproduction (Błoszyk et al. 2004). The insignificant influence of soil mean temperature on females recorded in our experiment, may be related to their year-round occurrence, in contrast to male mites, as well as to the reproductive strategy of a given gamasid species that creates soil mite communities.

## Conclusions

We found a significant influence of habitat type on gamasid mite assemblages of females, males and juvenile mites. Additionally, soil mean temperature had a significant effect on male and juvenile mites, but not on gamasid females. Moreover, litter type had a significant influence on mean juvenile abundance, whereas percentage litter mass loss significantly affected the female and juvenile developmental stages. However, taking habitat type into account, the percentage litter mass loss significantly affected female and male mites, but not the juvenile stages. Mite communities (female, male and juvenile instars) colonized litter extensively as decomposition progressed. The abundance calculated per 100 g dry litter mass usually increased gradually during decomposition—it had lower values at the beginning and the highest values at the last collection dates. The highest mean female, male and juvenile abundances were recorded in birch stands growing on the forest area; however, juvenile mites were also highly numerous in mixed stands on the spoil heap. All of the mesostigmatid developmental stages studied were the least abundant in monoculture stands of Scots pine growing on the spoil heap in comparison to other habitat types studied. We also found that male and juvenile mean abundances were highest in *P. tremula* litter, whereas females were highest in *U. laevis*. Furthermore, the lowest mean female and male abundances were recorded in *Q. robur* litter, whereas juvenile mites were lowest in *F. sylvatica* litter.

Our results confirm that mixed stands on post-mining areas are a potentially better habitat for mesostigmatid community development compared to monocultures, among others by relatively higher humidity and lower temperatures. Taking into account that the number of juvenile instars ensures the stability of a population and its possible development, it is important to properly shape and revitalize the degraded environment in order to improve the living conditions for Mesostigmata mite populations.

## Appendix 1: Checklist of gamasid species recorded from litterbags on 11 research plots within habitat types

**Table:**

No.	Mite species	Habitat type										
		FHbirch		FHpine		SHmixed			SHbirch		SHpine	
		1	2	3	4	5	6	7	8	9	10	11
1	*Trachytes aegrota* (C. L. Koch, 1841)	+++	+++	+	++	+++	+++	+++			+	+
2	*Veigaia nemorensis* (C. L. Koch, 1839)	+++	+++	++	++	+++	+++	+++			+	
3	*Zercon peltatus* C. L. Koch, 1836	+++	+++	+++	+++	+			+	+	+	+
4	*Olodiscus minima* (Kramer, 1882)	++	+++			+++	+++	++		+	+	+
5	*Gamasellodes bicolor* (Berlsese, 1918)	+		+	+	+++	+++	+++				
6	*Paragamasus runcatellus* (Berlese, 1903 sensu Karg, 1971)	++	++	+	++	+++	+++	+++	+	+	+	
7	*Amblyseius tubae* Karg, 1970	++	++	+	+				+++	+++	++	++
8	*Veigaia cerva* (Kramer, 1876)	++	++	++	++	+++	+++	++	++	+		
9	*Paragamasus* sp.	+	++	++	++	+++	+++	++	+	+	+	
10	*Pergamasus* sp.	++	++	++	++	+++	+++	++	+	++	++	++
11	*Pergamasus crassipes* (Linnaeus, 1758)	+	+	+	+	+++	+++	+++	++	+	++	++
12	*Vulgarogamasus kraepelini* (Berlese, 1904)	+				+++	+++	++				
13	*Amblyseius* sp.			+	+	++	++	+++	++	++	+	+
14	*Asca aphidioides* (Linnaeus, 1758)	++	++	++	++	++		+	++	++	++	++
15	*Paragamasus conus* (Karg, 1971)	+	+	+	+	+++	+++	+	+	+		
16	*Prozercon traegardhi* (Halbert, 1923)		+			++	++	+				
17	*Asca bicornis* (Canestrini & Fanzago, 1887)		+			+	+	+	++	++	++	++
18	*Pergamasus septentrionalis* (Oudemans, 1902)	++	++			++	++	++	+	+		
19	*Paragamasus jugincola* Athias-Henriot, 1967	++	++	+		++	++	++				
20	*Veigaia kochi* (Trägårdh, 1901)		+			++	++	++				
21	*Leptogamasus parvulus* (Berlese, 1903)	+	+	++	++				+			+
22	*Dendrolaelaps cornutus *sensu lato (Kramer, 1886)					+	++	++				
23	*Veigaia exigua* (Berlsese, 1916)					++	++	+				
24	*Discourella modesta* (Leonardi, 1889)					+	++	+				
25	*Arctoseius insularis* (Willmann, 1952)					+	+		+	+	++	++
26	*Veigaia planicola* (Berlese, 1892)	++										
27	*Oodinychus ovalis* (C. L. Koch, 1839)	+	+	+	+	+	++	+				
28	*Prozercon kochi* Sellnick, 1943			++								
29	*Iphidozercon gibbus* Berlese, 1903	+	++			+						
30	*Hypoaspis aculeifer* (Canestrini, 1883)					+	++	+				
31	*Arctoseius semiscissus* (Berlese, 1892)					++	+	+				
32	*Parazercon radiatus* (Berlese, 1914)			++			+					
33	*Hypoaspis praesternalis* Willmann, 1949				+		+		+	+	+	
34	*Hypoaspis vacua* (Michael, 1891)	+			+	+	+	+				
35	*Epicriopsis horridus* Kramer, 1876	+	++									
36	*Macrocheles montanus* (Willmann, 1951)	+	+	+								
37	*Dendrolaelaps angulosus* (Willmann, 1936)								+			+
38	*Dendrolaelaps foveolatus* (Leitner, 1949)											+
39	*Geholaspis longispinosus* (Kramer, 1876)		+									
40	*Dendrolaelaps punctatosimilis* Hirschmann, 1960	+	+						+	+		
41	*Eulaelaps stabularis* (C. L. Koch, 1839)					+						
42	*Eviphis ostrinus* (C. L. Koch, 1836)	+				+	+					
43	*Leptogamasus belligerens* Witaliński, 1973				+							
44	*Pergamasus barbarus* (Berlese, 1904)	+										
45	*Pergamasus brevicornis* (Berlese, 1903)		+	+								
46	*Dendrolaelaps rotundus* Hirschmann, 1960											+
47	*Lasioseius muricatus* (C. L. Koch, 1839)			+		+	+	+				
48	*Pergamasus mediocris* Berlese, 1904			+	+							
49	*Veigaia decurtata* Athias-Henriot, 1961					+	+					
50	*Laelaspis astronomicus* (C. L. Koch, 1839)				+							
51	*Alliphis halleri* (Canestrini & Canestrini, 1881)		+					+				
52	*Ameroseius corbiculus* (Sowerby, 1806)							+				
53	*Ameroseius pseudoplumosus* (Rack, 1972)							+				
54	*Antennoseius bacatus* Athias-Henriot, 1961										+	
55	*Antennoseius masoviae* Sellnick, 1943					+						+
56	*Dendrolaelaps latior* (Leitner, 1949)	+							+			
57	*Lasioseius ometes* (Oudemans, 1903)						+	+				
58	*Leioseius elongatus* Evans, 1958	+					+					
59	*Leptogamasus suecicus* (Trägårdh, 1936)						+					
60	*Proctolaelaps fisieri* Samšiňák, 1960					+						
61	*Ameroseius* sp.							+				
62	*Arctoseius brevichelis* Karg, 1969									+		
63	*Arctoseius venustulus* (Berlese, 1917)						+					
64	*Cheiroseius borealis* (Berlese, 1904)									+		
65	*Dendrolaelaps arvicolus* (Leitner, 1949)							+				
66	*Dendrolaelaps laetus* Shcherbak, 1980		+									
67	*Dendrolaelaps schauenburgi* (Schweizer, 1961)					+						
68	*Dermanyssus gallinae* (De Geer, 1778)						+					
69	*Dinychus perforatus* Kramer, 1882		+									
70	*Urobovella pulchella* (Berlese, 1904)		+									
71	*Lasioseius confusus* Evans, 1958			+								
72	*Lasioseius formosus* Westerboer, 1963					+						
73	*Leptogamasus anoxygenenellus* (Micherdziński, 1969)				+							
74	*Nejordensia levis* (Oudemans et Voigts, 1904)									+		
75	*Olodiscus misella* (Berlese, 1916)						+					
76	*Rhodacarellus silesiacus* Willmann, 1936							+				
77	*Sejus togatus* C. L. Koch, 1836			+								
78	*Typhlodromus pyri* Scheuten, 1857				+							
79	*Urodiaspis tecta* (Kramer, 1876)					+						
80	*Veigaia propinqua* Willmann, 1956						+					
81	*Zerconopsis muestairi* (Schweizer, 1949)											+

Symbols indicate abundance: +, 1–9 individuals; ++ 10–99; +++ >100. Mite species are ordered by total dominance

*FHbirch* monoculture stands of silver birch growing on forest habitat, *FHpine* monoculture stands of Scots pine growing on forest habitat, *SHmixed* mixed stands growing on spoil heap, *SHbirch* monoculture stands of silver birch growing on spoil heap, *SHpine* monoculture stands of Scots pine growing on spoil heap

## Appendix 2: Mean (± SD) female, male and juvenile abundances per 100 g dry litter mass within habitat types recorded for each collection date (interval day) since the start of the experiment

**Table:**

Habitat type	Interval day	Female	Male	Juvenile
FHbirch	91	45.09 ± 1.23	143.33 ± 26.01	55.21 ± 6.71
	182	167.92 ± 95.51	12.24 ± 9.44	164.67 ± 73.97
	275	683.73 ± 188.87	19.12 ± 14.41	319.26 ± 70.27
	366	1121.28 ± 294.63	129.52 ± 58.79	374.03 ± 61.28
	548	348.96 ± 36.95	34.98 ± 16.33	367.94 ± 202.19
	730	581.30 ± 3.93	227.06 ± 123.42	273.7 ± 14.85
	913	247.96 ± 201.16	16.97 ± 15.05	147.40 ± 150.18
	1100	1204.44 ± 416.25	140.30 ± 52.85	721.93 ± 94.02
FHpine	91	0	0	1.52 ± 2.15
	182	63.31 ± 89.53	4.04 ± 5.71	5.82 ± 8.23
	275	446.40 ± 16.51	8.49 ± 0.26	70.37 ± 38.37
	366	64.73 ± 2.76	33.86 ± 10.03	205.62 ± 15.75
	548	186.35 ± 42.88	46.22 ± 6.44	98.05 ± 8.80
	730	191.03 ± 135.14	58.70 ± 51.50	189.98 ± 15.30
	913	3.62 ± 2.64	0	0.82 ± 1.16
	1100	133.33 ± 52.75	33.96 ± 33.25	213.1 ± 25.72
SHmixed	95	19.16 ± 15.34	1.67 ± 2.90	2.68 ± 2.32
	196	153.65 ± 106.03	13.38 ± 8.59	248.02 ± 205.74
	285	224.72 ± 115.89	15.90 ± 6.06	248.50 ± 183.09
	385	148.10 ± 40.98	7.07 ± 2.54	53.34 ± 45.61
	564	175.88 ± 136.55	15.87 ± 10.68	508.52 ± 508.83
	753	208.82 ± 177.90	16.22 ± 9.97	117.59 ± 144.38
	936	187.92 ± 90.02	4.96 ± 4.63	574.36 ± 422.52
	1117	155.79 ± 58.25	22.73 ± 10.23	147.33 ± 157.16
	1298	162.14 ± 98.56	19.42 ± 9.30	121.63 ± 86.84
	1754	740.41 ± 471.20	44.70 ± 30.13	106.32 ± 42.83
SHbirch	91	0	0	0.65 ± 0.93
	182	0	0	0
	275	307.10 ± 32.01	53.20 ± 27.97	43.55 ± 16.81
	366	152.92 ± 40.79	8.59 ± 5.74	3.69 ± 0.90
	548	10.93 ± 4.92	11.44 ± 6.80	3.22 ± 2.41
	730	317.28 ± 309.24	4.22 ± 1.04	2.75 ± 1.44
	913	6.05 ± 8.55	5.35 ± 1.14	6.16 ± 8.71
	1100	162.43 ± 66.69	1.16 ± 1.64	5.76 ± 3.27
SHpine	91	0.79 ± 1.11	0	0.75 ± 1.06
	182	0	0	0
	275	70.30 ± 10.84	21.88 ± 1.19	40.02 ± 15.25
	366	34.06 ± 22.45	7.44 ± 3.88	2.81 ± 3.98
	548	18.22 ± 14.57	3.81 ± 3.78	4.62 ± 2.27
	730	45.74 ± 15.56	4.64 ± 6.57	1.03 ± 1.45
	913	3.65 ± 5.16	1.22 ± 1.72	1.22 ± 1.72
	1100	20.02 ± 9.82	12.74 ± 5.06	14.61 ± 15.83

*FHbirch* monoculture stands of silver birch growing on forest habitat, *FHpine* monoculture stands of Scots pine growing on forest habitat, *SHmixed* mixed stands growing on spoil heap, *SHbirch* monoculture stands of silver birch growing on spoil heap, *SHpine* monoculture stands of Scots pine growing on spoil heap

## Appendix 3: Mean (± SD) female, male and juvenile abundances per 100 g dry litter mass within 14 litter types recorded for each collection date (interval day) since the start of the experiment

**Table:**

Litter type	Interval day	Female	Male	Juvenile
Ace_pse	95	21.97 ± 18.25	1.25 ± 2.17	6.04 ± 7.50
	196	445.68 ± 223.06	64.77 ± 71.64	421.52 ± 383.11
	285	303.04 ± 91.14	8.80 ± 5.73	348.99 ± 224.22
	385	256.16 ± 179.81	15.24 ± 19.70	108.64 ± 145.65
	564	264.30 ± 293.83	69.75 ± 41.75	638.70 ± 592.07
	753	293.86 ± 176.27	24.79 ± 16.66	385.72 ± 475.08
	936	241.22 ± 132.22	17.86 ± 30.94	436.03 ± 163.21
	1117	244.84 ± 314.89	77.70 ± 134.58	745.74 ± 1291.66
	1298	343.34 ± 122.61	26.65 ± 46.15	268.26 ± 140.57
	1754	645.52 ± 478.74	0	189.10 ± 183.53
Aln_glu	95	24.84 ± 21.92	1.88 ± 3.25	3.67 ± 4.20
	196	281.85 ± 249.64	11.36 ± 10.44	542.98 ± 473.00
	285	254.83 ± 135.75	20.31 ± 17.81	379.65 ± 312.31
	385	195.77 ± 57.90	10.61 ± 4.14	106.26 ± 99.04
	564	236.19 ± 238.20	24.24 ± 23.32	861.82 ± 1167.16
	753	264.23 ± 376.36	10.53 ± 9.44	57.29 ± 99.24
	936	94.24 ± 82.64	0	968.95 ± 816.75
	1117	52.88 ± 45.82	0	46.03 ± 79.72
	1298	118.10 ± 50.66	0	69.73 ± 33.98
	1754	1030.30 ± 424.53	114.60 ± 105.92	105.37 ± 42.57
Bet_pen	95	23.25 ± 9.67	2.46 ± 4.27	1.21 ± 2.09
	196	70.45 ± 42.84	11.09 ± 8.98	84.54 ± 72.65
	285	266.85 ± 173.28	17.32 ± 8.12	210.64 ± 117.71
	385	118.32 ± 34.42	11.91 ± 5.05	7.81 ± 6.86
	564	135.62 ± 97.91	16.29 ± 15.39	367.53 ± 305.70
	753	208.31 ± 23.50	20.36 ± 18.59	136.33 ± 110.04
	936	366.70 ± 245.34	12.50 ± 11.06	613.13 ± 773.04
	1117	326.72 ± 137.94	74.20 ± 33.81	238.85 ± 237.89
	1298	141.09 ± 176.73	30.63 ± 29.09	111.69 ± 96.76
	1754	1119.86 ± 1635.19	4.73 ± 8.20	47.15 ± 34.79
Fag_syl	95	17.43 ± 12.68	3.50 ± 6.06	3.48 ± 3.52
	196	140.42 ± 47.49	28.25 ± 15.94	134.20 ± 99.00
	285	181.52 ± 41.18	38.97 ± 22.28	133.73 ± 109.80
	385	154.65 ± 90.49	10.60 ± 12.29	24.69 ± 13.81
	564	54.63 ± 51.25	27.83 ± 48.21	92.76 ± 87.77
	753	87.50 ± 75.94	7.10 ± 8.82	16.73 ± 21.55
	936	90.73 ± 85.86	7.69 ± 3.04	197.40 ± 182.57
	1117	100.27 ± 20.59	1.71 ± 2.96	84.78 ± 72.71
	1298	212.02 ± 192.33	25.10 ± 19.10	193.84 ± 171.94
	1754	162.34 ± 110.46	15.39 ± 26.65	104.47 ± 40.32
Fra_exc	95	38.61 ± 44.96	6.55 ± 5.89	6.59 ± 5.92
	196	182.59 ± 84.79	14.39 ± 8.25	173.80 ± 137.39
	285	392.44 ± 303.38	40.41 ± 14.44	559.46 ± 466.35
	385	281.31 ± 160.82	15.29 ± 26.48	41.39 ± 42.63
	564	157.61 ± 160.55	17.16 ± 15.13	511.10 ± 652.67
	753	486.05 ± 205.55	23.46 ± 22.66	343.12 ± 345.43
	936	279.20 ± 310.16	12.73 ± 22.05	931.80 ± 1046.66
	1117	209.96 ± 184.37	0	73.33 ± 115.26
	1298	388.20 ± 20.99	74.80 ± 74.44	167.55 ± 128.70
	1754	592.11 ± 136.74	0	196.53 ± 126.75
Pin_syl	95	9.76 ± 6.63	1.76 ± 3.05	3.57 ± 3.10
	196	139.37 ± 81.58	17.12 ± 13.17	168.66 ± 169.00
	285	163.83 ± 93.51	12.98 ± 7.49	204.09 ± 235.06
	385	132.01 ± 79.98	0	54.99 ± 44.57
	564	201.38 ± 193.61	9.36 ± 4.81	357.20 ± 319.09
	753	215.83 ± 141.35	25.52 ± 22.98	178.27 ± 218.80
	936	174.75 ± 48.98	3.69 ± 6.40	356.84 ± 245.18
	1117	98.87 ± 41.45	1.73 ± 3.00	173.39 ± 233.17
	1298	231.81 ± 92.09	18.34 ± 10.91	165.04 ± 115.87
	1754	291.84 ± 139.28	34.80 ± 29.72	135.08 ± 58.67
Pop_can	95	58.81 ± 14.21	9.27 ± 3.09	8.20 ± 4.77
	196	187.08 ± 167.86	12.63 ± 11.39	192.28 ± 141.01
	285	245.37 ± 217.23	11.53 ± 13.12	241.39 ± 154.13
	385	174.27 ± 21.18	5.24 ± 5.71	74.36 ± 89.78
	564	149.83 ± 139.82	17.31 ± 14.04	403.89 ± 578.32
	753	246.33 ± 50.54	15.83 ± 27.42	165.75 ± 141.76
	936	14.11 ± 6.31	0	72.77 ± 72.94
	1117	405.62 ± 552.54	93.70 ± 162.30	772.28 ± 1337.63
	1298	211.01 ± 114.05	0	261.96 ± 317.30
	1754	1755.84 ± 1995.99	23.11 ± 24.52	192.59 ± 108.42
Pop_nig	95	19.50 ± 15.79	0	5.38 ± 5.37
	196	96.97 ± 34.46	13.57 ± 5.10	361.05 ± 190.78
	285	222.34 ± 100.10	20.64 ± 17.06	181.18 ± 116.67
	385	106.02 ± 35.97	8.37 ± 7.94	16.81 ± 21.12
	564	88.14 ± 85.25	6.98 ± 8.02	274.51 ± 92.30
	753	127.61 ± 167.60	9.91 ± 17.17	97.46 ± 111.07
	936	100.80 ± 79.60	4.84 ± 4.33	207.88 ± 290.58
	1117	159.66 ± 92.48	2.62 ± 4.53	52.66 ± 82.75
	1298	192.37 ± 91.78	30.29 ± 17.26	133.99 ± 106.34
	1754	435.25 ± 236.52	8.17 ± 7.53	116.38 ± 59.28
Pop_tre	95	14.38 ± 7.29	1.16 ± 2.01	4.70 ± 5.33
	196	276.14 ± 115.48	34.47 ± 22.75	222.94 ± 157.86
	285	384.09 ± 232.81	13.05 ± 8.18	232.87 ± 189.73
	385	240.57 ± 125.45	9.51 ± 8.80	38.39 ± 11.78
	564	185.57 ± 144.54	3.19 ± 5.53	463.29 ± 472.36
	753	277.55 ± 95.83	12.95 ± 22.44	49.86 ± 39.39
	936	209.92 ± 221.63	5.73 ± 9.93	1882.51 ± 2544.38
	1117	254.14 ± 55.07	48.31 ± 83.67	71.94 ± 71.68
	1298	300.84 ± 340.74	25.08 ± 22.02	1355.94 ± 1862.42
	1754	1054.16 ± 1185.04	383.14 ± 663.62	797.58 ± 1091.77
Pru_ser	95	40.10 ± 39.22	0	4.43 ± 4.75
	196	181.20 ± 163.63	23.13 ± 25.10	145.18 ± 137.40
	285	242.23 ± 236.27	29.27 ± 42.29	336.28 ± 347.47
	385	280.76 ± 29.43	4.59 ± 7.95	33.33 ± 30.95
	564	480.32 ± 483.45	7.98 ± 13.83	1078.41 ± 1441.01
	753	510.25 ± 390.21	73.77 ± 67.44	460.36 ± 286.27
	936	0	0	437.81 ± 758.32
	1117	722.34 ± 1190.74	165.84 ± 287.24	2172.53 ± 3762.94
	1298	96.15 ± 83.28	0	69.20 ± 67.52
	1754	164.51 ± 158.67	14.15 ± 24.52	260.50 ± 68.84
Que_rob	95	12.01 ± 20.80	0	1.59 ± 2.76
	196	58.13 ± 29.21	17.02 ± 12.74	60.35 ± 52.48
	285	211.86 ± 234.45	9.08 ± 11.40	125.12 ± 176.07
	385	122.27 ± 58.32	7.32 ± 3.08	9.14 ± 11.30
	564	60.84 ± 50.32	11.42 ± 10.01	310.55 ± 309.88
	753	84.77 ± 91.02	2.80 ± 4.86	88.74 ± 139.39
	936	44.04 ± 41.63	2.36 ± 4.09	142.66 ± 141.39
	1117	133.58 ± 50.40	7.22 ± 6.26	114.78 ± 93.68
	1298	152.96 ± 128.37	38.03 ± 35.45	158.52 ± 148.68
	1754	298.89 ± 249.75	4.61 ± 7.99	169.06 ± 280.92
Que_rub	95	20.58 ± 9.02	0	3.42 ± 5.93
	196	206.54 ± 163.57	20.89 ± 10.37	168.41 ± 144.94
	285	294.59 ± 254.73	20.96 ± 9.29	187.38 ± 171.57
	385	144.55 ± 44.89	10.38 ± 9.18	25.37 ± 30.51
	564	128.29 ± 106.67	13.58 ± 7.22	163.60 ± 154.52
	753	224.31 ± 228.58	0	180.66 ± 302.47
	936	130.06 ± 86.53	2.59 ± 4.48	213.58 ± 100.18
	1117	239.58 ± 184.76	20.29 ± 19.31	236.09 ± 246.51
	1298	183.62 ± 151.44	5.06 ± 8.77	305.40 ± 129.60
	1754	351.45 ± 263.83	25.16 ± 22.17	51.46 ± 41.62
Rob_pse	95	37.05 ± 0.55	6.22 ± 4.13	8.68 ± 5.81
	196	70.23 ± 17.45	11.65 ± 7.43	205.66 ± 154.63
	285	178.87 ± 96.61	8.64 ± 7.49	143.10 ± 130.49
	385	143.97 ± 68.64	15.06 ± 9.31	10.78 ± 7.52
	564	112.61 ± 97.77	16.08 ± 18.02	195.10 ± 211.20
	753	156.37 ± 17.53	12.18 ± 1.60	45.35 ± 10.77
	936	135.45 ± 112.16	13.66 ± 16.41	175.49 ± 191.75
	1117	180.16 ± 138.03	7.27 ± 6.31	165.20 ± 116.85
	1298	328.56 ± 208.11	43.10 ± 21.51	182.37 ± 114.74
	1754	262.55 ± 141.32	61.90 ± 13.25	110.34 ± 66.26
Ulm_lae	95	32.94 ± 15.33	5.48 ± 1.96	5.29 ± 1.84
	196	240.40 ± 141.99	25.92 ± 3.88	264.31 ± 192.76
	285	217.50 ± 161.30	11.27 ± 7.75	317.28 ± 333.74
	385	256.98 ± 218.95	13.71 ± 12.61	139.88 ± 150.26
	564	94.25 ± 82.15	3.80 ± 3.34	454.73 ± 388.49
	753	782.74 ± 715.41	43.05 ± 43.83	510.67 ± 451.61
	936	194.11 ± 336.21	78.05 ± 85.95	529.06 ± 730.71
	1117	359.17 ± 439.75	15.52 ± 26.88	37.90 ± 33.42
	1298	1700.78 ± 2140.56	0	285.76 ± 168.39
	1754	1348.60 ± 890.47	6.57 ± 11.37	761.91 ± 681.43

Litter types are abbreviated to the first three letters of genera name and first three letters of the species name

## References

[CR1] Alberti G, di Palma A, Krantz GW, Błaszak C, Sabelis MW, Bruin J (2010). First ultrastructural observations on a putative sperm access system in veigaiid females (Veigaiidae, Gamasida). Trends in acarology.

[CR2] Azevedo LH, Ferreira MP, de Castilho RC (2018). Potential of *Macrocheles species* (Acari: Mesostigmata: Macrochelidae) as control agents of harmful flies (Diptera) and biology of *Macrocheles embersoni* Azevedo, Castilho and Berto on *Stomoxys calcitrans* (L.) and *Musca domestica* L. (Diptera: Muscidae). Biol Control.

[CR3] Bates D, Mächler M, Bolker B, Walker S (2015). Fitting linear mixed-effects models using lme4. J Stat Softw.

[CR4] Beaulieu F, Weeks AR (2007). Free-living mesostigmatic mites in Australia: their roles in biological control and bioindication. Aust J Exp Agric.

[CR5] Berg B, McClaugherty C (2014). Plant litter, decomposition, humus formation, carbon sequestration.

[CR6] Błoszyk J, Adamski Z, Napierała A, Dylewska M (2004). Parthenogenesis as a life strategy among mites of the suborder Uropodina (Acari: Mesostigmata). Can J Zool.

[CR7] Bogdanowicz W, Chudzicka E, Pilipiuk I, Skibińska E (2008). Fauna of Poland—characteristics and checklist of species.

[CR8] Brückner A, Klompen H, Bruce AI (2017). Infection of army ant pupae by two new parasitoid mites (Mesostigmata: Uropodina). PeerJ.

[CR9] Cavard X, Macdonald SE, Bergeron Y, Chen HYH (2011). Importance of mixedwoods for biodiversity conservation: evidence for understory plants, songbirds, soil fauna, and ectomycorrhizae in northern forests. Environ Rev.

[CR10] Cesarz S, Fahrenholz N, Migge-Kleian S (2007). Earthworm communities in relation to tree diversity in a deciduous forest. Eur J Soil Biol.

[CR11] Chaturvedi RK, Singh JS (2017). Restoration of mine spoil in a dry tropical region: a review. Proc Indian Natl Sci Acad USA.

[CR12] de Mendiburu F (2013) Statistical procedures for agricultural research. Package ‘Agricolae,’ version 1.4-4. Comprehensive R Archive Network, Institute for Statistics and Mathematics, Vienna, Austria

[CR13] Dyderski MK, Jagodziński AM (2019). Seedling survival of *Prunus serotina* Ehrh., *Quercus rubra* L. and *Robinia pseudoacacia* L. in temperate forests of Western Poland. For Ecol Manag.

[CR14] Eisenhauer N, Antunes PM, Bennett AE (2017). Priorities for research in soil ecology. Pedobiologia.

[CR15] Elmer M, La France M, Förster G, Roth M (2004). Changes in the decomposer community when converting spruce monocultures to mixed spruce/beech stands. Plant Soil.

[CR16] FAO, ITPS, GSBI, CBD and EC (2020). State of knowledge of soil biodiversity—status, challenges and potentialities: Report 2020.

[CR17] Field CB, Behrenfeld MJ, Randerson JT, Falkowski P (1998). Primary production of the biosphere: integrating terrestrial and oceanic components. Science.

[CR18] Frouz J (2018). Effects of soil macro- and mesofauna on litter decomposition and soil organic matter stabilization. Geoderma.

[CR19] Fujii S, Takeda H (2017). Succession of soil microarthropod communities during the aboveground and belowground litter decomposition processes. Soil Biol Biochem.

[CR20] Giljarov MS, Bregetova NG (1977). A key to soil-inhabiting mites, Mesostigmata.

[CR21] Graca MAS, Bärlocher F, Gessner MO (2005). Methods to study litter decomposition. A practical guide.

[CR22] Gwiazdowicz DJ (2007) Ascid mites (Acari. Mesostigmata) from selected forest ecosystems and microhabitats in Poland. Akademia Rolnicza im. Augusta Cieszkowskiego, Poznań

[CR23] Hasegawa M, Okabe K, Fukuyama K (2013). Community structures of Mesostigmata, Prostigmata and Oribatida in broad-leaved regeneration forests and conifer plantations of various ages. Exp Appl Acarol.

[CR24] Horodecki P, Jagodziński AM (2019). Site type effect on litter decomposition rates: a three-year comparison of decomposition process between spoil heap and forest sites. Forests.

[CR25] Horodecki P, Nowiński M, Jagodziński AM (2019). Advantages of mixed tree stands in restoration of upper soil layers on postmining sites: a five-year leaf litter decomposition experiment. Land Degrad Dev.

[CR26] Huhta V, Hänninen S-M (2001). Effects of temperature and moisture fluctuations on an experimental soil microarthropod community. Pedobiologia.

[CR27] Irmler U (2000). Changes in the fauna and its contribution to mass loss and N release during leaf litter decomposition in two deciduous forests. Pedobiologia.

[CR28] Jagodziński AM, Wierzcholska S, Dyderski MK (2018). Tree species effects on bryophyte guilds on a reclaimed post-mining site. Ecol Eng.

[CR29] Kamczyc J, Dyderski MK, Horodecki P, Jagodziński AM (2019). Mite communities (Acari, Mesostigmata) in the initially decomposed ‘litter islands’ of 11 tree species in scots pine (*Pinus sylvestris* L.) forest. Forests.

[CR30] Kampichler C, Bruckner A (2009). The role of microarthropods in terrestrial decomposition: a meta-analysis of 40 years of litterbag studies. Biol Rev.

[CR31] Karg W (1971) Acari (Acarina) Milben, Unterordnung Anactinochaeta (Parasitiformes). Die freilebenden Gamasina (Gamasides). Raubmilben, Die Tierwelt Deutschlands 59. Gustav Fischer Verlag, Jena

[CR32] Karg W (1993) Acari (Acarina) Milben Parasitiformes (Anactinochaeta), Cohors Gamasina Leach Raubmmilben. Die Tierwelt Deutschlands. VEB Gustav Fischer Verlag, Jena

[CR33] Koehler HH (1997). Mesostigmata (Gamasina, Uropodina), efficient predators in agroecosystems. Agric Ecosyst Environ.

[CR34] Koehler HH (1999). Predatory mites (Gamasina, Mesostigmata). Agric Ecosyst Environ.

[CR35] Korboulewsky N, Perez G, Chauvat M (2016). How tree diversity affects soil fauna diversity: a review. Soil Biol Biochem.

[CR36] Kuznetsova A, Brockhoff PB, Christensen RHB (2017). lmerTest Package: tests in linear mixed effects models. J Stat Softw.

[CR37] Lawrence KL, Wise DH (2000). Spider predation on forest-floor Collembola and evidence for indirect effects on decomposition. Pedobiologia.

[CR38] Lawrence KL, Wise DH (2004). Unexpected indirect effect of spiders on the rate of litter disappearance in a deciduous forest. Pedobiologia.

[CR39] Lorenz K, Preston CM, Krumrei S, Feger K-H (2004). Decomposition of needle/leaf litter from Scots pine, black cherry, common oak and European beech at a conurbation forest site. Eur J for Res.

[CR40] Madej G (2002). Roztocze Mesostigmata (Arachnida, Acari) jako dobry wskaźnik stadiów sukcesyjnych hałd [Soil mesostigmatid mites (Arachnida, Acari) as a good indicator of succession stages on dumps]. Kosmos.

[CR41] Madej G (2004) Rozwój zgrupowań roztoczy Mesostigmata (Arachnida. Acari) na nieużytkach poprzemysłowych [Development of communities of mesostigmatid mite (Arachnida, Acari) in areas of postindustrial wastelands]. Uniwersytet Śląski, Katowice

[CR42] Madej G, Skubała P (1996). Communites of mites (Acari) on old galena-calamine mining wastelands at Galman, Poland. Pedobiologia.

[CR43] Madej G, Skubała P, Bernini F, Nannelli R, Nuzzaci G, de Lillo E (2002). Colonization of a dolomitic dump by mesostigmatid mites (Acari, Mesostigmata). Acarid phylogeny and evolution: adaptation in mites and ticks.

[CR44] Maraun M, Scheu S (2000). The structure of oribatid mite communities (Acari, Oribatida): patterns, mechanisms and implications for future research. Ecography.

[CR45] Micherdziński W (1969) Die Familie Parasitidae Oudemans. 1901 (Acarina, Mesostigmata). PWN, Kraków

[CR46] Miller TEX, Rudolf VHW (2011). Thinking inside the box: community-level consequences of stage-structured populations. Trends Ecol Evol.

[CR47] Mueller KE, Eisenhauer N, Reich PB (2016). Light, earthworms, and soil resources as predictors of diversity of 10 soil invertebrate groups across monocultures of 14 tree species. Soil Biol Biochem.

[CR48] Murvanidze M, Mumladze L, Arabuli T, Kvavadze E (2013). Oribatid mite colonization of sand and manganese tailing sites. Acarologia.

[CR49] Mwabvu T (2014). Surface-active millipedes (Diplopoda) and associated mites (Acari, Mesostigmata) in Pigeon Valley Nature Reserve in Durban, South Africa. Soil Organisms.

[CR50] Napierała A, Błoszyk J (2013). Unstable microhabitats (merocenoses) as specific habitats of Uropodina mites (Acari: Mesostigmata). Exp Appl Acarol.

[CR51] Neuner S, Albrecht A, Cullmann D (2015). Survival of Norway spruce remains higher in mixed stands under a dryer and warmer climate. Glob Chang Biol.

[CR52] Norton RA, Kethley JB, Johnston DE, O’Connor BM, Wrensch D, Ebbert MA (1993). Phylogenetic perspectives on genetic systems and reproductive modes of mites. Evolution and diversity of sex ratio in insects and mites.

[CR53] Oliveira DGPD, Kasburg CR, Alves LFA (2020). Efficacy of *Beauveria bassiana* against the poultry red mite, *Dermanyssus gallinae* (De Geer, 1778) (Mesostigmata: Dermanyssidae), under laboratory and hen house conditions. Syst Appl Acarol.

[CR54] Osler GHR, Gauci CS, Abbott LK (2004). Limited evidence for short-term succession of microarthropods during early phases of surface litter decomposition. Pedobiologia.

[CR55] Pastwik E, Skorupski M, Piasta A, Jagodziński AM (2013) Mesostigmata mites of afforested post-industrial habitats on a lignite mine spoil heap in Bełchatów – a preliminary study. In: Neményi M, Varga L, Facskó F, Lőrincz I (eds) Science for sustainability. Proceedings of the International Scientific Conference for PhD Students. Győr, March 19–20, 2013. University of West Hungary Press, Sopron, pp 251–257

[CR56] Piasta A, Skorupski M, Horodecki P, Jagodziński AM (2015). Zgrupowania roztoczy (Acari) pod drzewostanami sosnowymi na terenach leśnych i rekultywowanym zwałowisku zewnętrznym w Nadleśnictwie Bełchatów [Soil mite communities under Scots pine stands growing on forest sites and reclaimed lignite mine spoil heap in Bełchatów Forest District]. Studia i Materiały Centrum Edukacji Przyrodniczo-Leśnej w Rogowie.

[CR57] Pietrzykowski M (2010). Scots pine (*Pinus sylvestris* L.) ecosystem macronutrients budget on reclaimed mine sites—stand trees supply and stability. Nat Sci.

[CR58] Pietrzykowski M, Daniels WL (2014). Estimation of carbon sequestration by pine (*Pinus sylvestris* L.) ecosystems developed on reforested post-mining sites in Poland on differing mine soil substrates. Ecol Eng.

[CR59] Polis GA, Strong DR (1996). Food web complexity and community dynamics. Am Nat.

[CR60] Rawlik M, Kasprowicz M, Jagodziński AM (2018). Differentiation of herb layer vascular flora in reclaimed areas depends on the species composition of forest stands. For Ecol Manag.

[CR61] Rawlik M, Kasprowicz M, Jagodziński AM (2018). Canopy tree species determine herb layer biomass and species composition on a reclaimed mine spoil heap. Sci Total Environ.

[CR62] Robakowski P, Bielinis E, Sendall K (2018). Light energy partitioning, photosynthetic efficiency and biomass allocation in invasive *Prunus serotina* and native *Quercus petraea* in relation to light environment, competition and allelopathy. J Plant Res.

[CR63] Roth MA, Wilson JM, Tignor KR, Gross AD (2020). Biology and management of *Varroa destructor* (Mesostigmata: Varroidae) in *Apis mellifera* (Hymenoptera: Apidae) colonies. J Integr Pest Manag.

[CR64] Rudolf VHW, Rasmussen NL (2013). Population structure determines functional differences among species and ecosystem processes. Nat Commun.

[CR65] Sako A, Semde S, Wenmenga U (2018). Geochemical evaluation of soil, surface water and groundwater around the Tongon gold mining area, northern Cote d’Ivoire, West Africa. J Afr Earth Sci.

[CR66] Salmane I, Spuņģis V (2015). Factors influencing Mesostigmata mites (Acari, Parasitiformes) in the alkaline fen habitats. Proc Latv Acad Sci Sect B.

[CR67] Schmidt RA (2014). Leaf structures affect predatory mites (Acari: Phytoseiidae) and biological control: a review. Exp Appl Acarol.

[CR68] Seniczak S, Seniczak A, Gwiazdowicz DJ, Coulson SJ (2014). Community structure of Oribatid and Gamasid mites (Acari) in Moss-Grass Tundra in Svalbard (Spitsbergen, Norway). Arct Antarct Alp Res.

[CR69] Seniczak S, Graczyk R, Seniczak A (2018). Microhabitat preferences of Oribatida and Mesostigmata (Acari) inhabiting lowland beech forest in Poland and the trophic interactions between these mites. Eur J Soil Biol.

[CR70] Schulze ED (2000). Carbon and nitrogen cycling in European forest ecosystems. Ecological studies.

[CR71] Skorupski M (2010). Influence of selected tree species on forest ecosystem biodiversity for the example of Mesostigmata mites in a common-garden experiment.

[CR72] Skorupski M, Horodecki P, Jagodziński AM (2013) Roztocze z rzędu Mesostigmata (Arachnida, Acari) na terenach przemysłowych i poprzemysłowych w Polsce [Mite species of Mesostigmata order (Arachnida, Acari) in industrial and postindustrial areas of Poland]. Nauka Przyroda Technologie 7:#11

[CR73] Skubała P, Bernini F, Nannelli R, Nuzzaci G, de Lillo E (2002). Development of oribatid mite communities (Acari, Oribatida) on a mine dump. Acarid phylogeny and evolution: adaptation in mites and ticks.

[CR74] Skubała P (2002). Rozwój fauny roztoczy na hałdach, czyli jak przyroda walczy z przemysłem [The development of mite fauna on dumps or how nature struggles with industry]. Kosmos.

[CR75] Thakur MP, Reich PB, Hobbie SE (2018). Reduced feeding activity of soil detritivores under warmer and drier conditions. Nat Clim Chang.

[CR76] Tuka E, Solarz K (2017). Dynamika sezonowa i struktura populacji roztoczy Dermatophagoides w kurzu z miejsc do spania na terenie Sosnowca [Seasonal dynamics and population structure of Dermatophagoides in dust from sleeping places in the area of Sosnowiec city]. Alergoprofil.

[CR77] Urbanowski CK, Horodecki P, Kamczyc J (2018). Succession of mite assemblages (Acari, Mesostigmata) during decomposition of tree leaves in forest stands growing on reclaimed post-mining spoil heap and adjacent forest habitats. Forests.

[CR78] Urbanowski CK, Horodecki P, Kamczyc J (2021). Does litter decomposition affect mite communities (Acari, Mesostigmata)? A five-year litterbag experiment with 14 tree species in mixed forest stands growing on a post-industrial area. Geoderma.

[CR79] Vacek Z, Prokůpková A, Vacek S (2021). Mixed vs. monospecific mountain forests in response to climate change: structural and growth perspectives of Norway spruce and European beech. For Ecol Manag.

[CR80] Vitali V, Forrester DI, Bauhus J (2018). Know Your neighbours: drought response of Norway spruce, silver fir and Douglas fir in mixed forests depends on species identity and diversity of tree neighbourhoods. Ecosystems.

[CR81] von Wendorff G (1952) Die *Prunus serotina* in Mitteleuropa. Eine waldbauliche Monographie. PhD Dissertation, Universitat Hamburg, Germany

[CR82] Vreeken-Buijs MJ, Brussaard L (1996). Soil mesofauna dynamics, wheat residue decomposition and nitrogen mineralization in buried litterbags. Biol Fertil Soils.

[CR83] Walter DE, Proctor HC (2013). Mites in soil and litter systems. Mites: ecology, evolution and behaviour.

[CR84] Wissuwa J, Salamon J-A, Frank T (2012). Effects of habitat age and plant species on predatory mites (Acari, Mesostigmata) in grassy arable fallows in Eastern Austria. Soil Biol Biochem.

